# LESSDD-Net: A Lightweight and Efficient Steel Surface Defect Detection Network Based on Feature Segmentation and Partially Connected Structures

**DOI:** 10.3390/s26030753

**Published:** 2026-01-23

**Authors:** Jiayu Wu, Longxin Zhang, Xinyi Pu

**Affiliations:** School of Computer Science and Artificial Intelligence, Hunan University of Technology, Zhuzhou 412007, China; wujiayu@stu.hut.edu.cn (J.W.); 22450150520@stu.hut.edu.cn (X.P.)

**Keywords:** attention mechanism, downsampling, lightweight, surface defect detection

## Abstract

Steel surface defect detection is essential for maintaining industrial production quality and operational safety. However, existing deep learning-based methods often encounter high computational costs, hindering their deployment on mobile devices. To effectively address this challenge, we propose a lightweight and efficient steel surface defect detection network based on feature segmentation and partially connected structures, termed LESSDD-Net. In LESSDD-Net, we first introduce a lightweight downsampling module called the cross-stage partial-based dual-branch downsampling module (CSPDDM). This module significantly reduces the number of model parameters and computational costs while facilitating more efficient downsampling operations. Next, we present a lightweight attention mechanism known as coupled channel attention (CCAttention), which enhances the model’s capability to capture essential information in feature maps. Finally, we improve the faster implementation of cross-stage partial bottleneck with two convolutions (C2f) and design a lightweight version called the lightweight and partial faster implementation of cross-stage partial bottleneck with two convolutions (LP-C2f). This module not only enhances detection accuracy but also further diminishes the model’s size. Experimental results on the data-augmented Northeastern University surface defect detection (NEU-DET) dataset indicate that the mean average precision (mAP) of LESSDD-Net improves by 3.19% compared to the baseline model YOLO11n. Additionally, in terms of model complexity, LESSDD-Net reduces the number of parameters and computational costs by 39.92% and 20.63%, respectively, compared to YOLO11n. When compared with other mainstream object detection models, LESSDD-Net achieves top detection accuracy with the highest mAP value and demonstrates significant advantages in model complexity, characterized by the lowest number of parameters and computational costs.

## 1. Introduction

As a cornerstone of the national economy, the steel industry plays a crucial role in supporting modern life and fostering economic growth [1]. The wide range of products produced by the steel industry has extensive applications in various sectors, such as infrastructure development, transportation, automotive manufacturing, and the aerospace industry [2]. Consequently, the quality and performance of steel have long been central concerns for both academic researchers and industry professionals. As manufacturing standards continue to evolve, the market’s demand for high-quality steel has become increasingly rigorous [3]. However, due to the intricate production processes and environmental factors, steel surfaces often exhibit a range of defects during both manufacturing and transportation [4]. These defects not only affect the material’s appearance and reliability but also shorten its service life. Additionally, they pose a risk of significant economic losses and could potentially lead to serious safety incidents [5]. To ensure production quality and enhance industrial efficiency, there is an urgent requirement for quick and accurate methods for detecting surface defects in steel.

In the initial phases of steel surface defect detection, manual visual inspection was the main approach for identifying surface flaws. However, this method is prone to subjective biases and inspector fatigue, leading to low detection efficiency and making it difficult to maintain consistent quality in detection [6].

In contrast to the limitations of traditional manual visual inspection, which include lengthy detection times and low reliability, machine vision-based defect detection methods have shown impressive capabilities in object classification [7,8]. However, this approach has inherent limitations as it depends on manually defined defect features. It demonstrates poor generalization because of its reliance on low-dimensional, manually defined feature representations, particularly when addressing complex and variable steel surface defects [9]. Moreover, extracting a large number of features can significantly slow down detection speed, hindering the practical application of machine vision-based defect detection in industrial settings [10].

In recent years, the rapid advancement of deep learning technology and computer vision has led to significant progress in object detection methods based on convolutional neural networks (CNNs) [11]. Depending on the detection method, deep learning-based object detection algorithms can be primarily categorized into two types: two-stage detection algorithms and one-stage detection algorithms [12]. Among these, faster regions with convolutional neural network [13] serve as a prominent example of a two-stage detection algorithm. By utilizing a fully convolutional network to develop the region proposal network, this method enables an end-to-end coupling of candidate region generation and feature extraction. This design incorporates the offline phase of traditional selective search into the neural network, allowing for the sharing of underlying convolutional computations between the region proposal and feature extraction stages. As a result, it significantly enhances inference efficiency while preserving high precision in localization. In contrast to the focus of two-stage detection algorithms on detection accuracy, one-stage detection algorithms manage to strike a balance between speed and accuracy by streamlining the workflow. This type of algorithm abandons explicit candidate region generation mechanisms, instead adopting a dense prediction strategy to directly obtain regression target bounding boxes and class probabilities on feature maps. Notable examples of this approach include the you only look once (YOLO) series [14] and the single shot multiBox detector (SSD) [15].

Among the many mainstream object detection algorithms, one-stage models, like the YOLO series, demonstrate significant performance advantages due to their effective balance between speed and accuracy [16,17]. As a key development in the ongoing evolution of the YOLO architecture, YOLO11 [18] enhances detection accuracy while preserving high inference speed. However, there is still potential for enhancement regarding model complexity and computational efficiency.

In the steel manufacturing industrial environment, the dual challenges of inherent scene characteristics and cost factors significantly impact the performance of steel surface defect detection equipment. As a result, current equipment struggles to meet the computational resource demands of large-scale detection models. From a practical standpoint, the steel production process encompasses multiple intricate stages, including ironmaking, steelmaking, continuous casting, and rolling, each capable of producing various types of surface defects. For instance, during the rolling process, issues such as roll wear, improper temperature control, or tension fluctuations can lead to defects like cracks, scratches, and patches on the steel surface. These defects vary in shape, size, and texture, and their occurrence is both random and uncertain. To accurately identify this diverse range of defects, large-scale detection models must analyze substantial amounts of image data and employ complex algorithms for feature extraction and classification, necessitating extensive computing resources [19]. Regarding cost factors, deploying and operating large-scale detection models entails expenses across multiple aspects. From a hardware perspective, high-end servers or specialized artificial intelligence accelerator cards are necessary to achieve the required high computing performance. The significant costs of these hardware components increase the initial investment for enterprises. Additionally, the operation of large-scale detection models consumes a substantial amount of electricity, and with rising energy prices, electricity costs have become a considerable financial burden for companies. Furthermore, to ensure the reliable operation of testing equipment, professional technicians must be employed for maintenance and management, which adds to labor costs. The complexity of real-world scenarios, combined with these cost constraints, results in notable deficiencies in the computing resources of current steel surface defect detection equipment. Under resource limitations, such equipment struggles to provide the necessary computing power to support the efficient functioning of large-scale detection models, leading to slow detection speeds and reduced accuracy, ultimately failing to meet the demands for high-quality and efficient detection in steel production.

Building on this foundation, this study enhances existing steel surface defect detection models based on YOLO11, addressing issues related to large parameter sizes and computational demands. It introduces a novel lightweight and efficient steel surface defect detection network, termed the LESSDD-Net, which utilizes feature segmentation and partially connected structures. This algorithm seeks to maintain high detection performance while minimizing model parameters and computational requirements, thereby increasing its applicability and generalization in industrial defect detection scenarios. The main contributions of this study are summarized as follows:

(1) We propose a lightweight downsampling module known as the cross-stage partial-based dual-branch downsampling module (CSPDDM). This module utilizes a dual-branch architecture to independently and synergistically process spatial and channel information. It effectively reduces the resolution of feature maps while maximizing the preservation of both shallow-level detail and deep-level semantic information. This approach facilitates the efficient extraction of key feature information while significantly decreasing the model’s parameter count and computational complexity.
(2) We introduce a lightweight attention mechanism called coupled channel attention (CCAttention) as a replacement for the cross-stage partial with pyramid squeeze attention in the YOLO11 backbone network. CCAttention is a coordinate-aware coupled channel attention mechanism that employs zero-space parameters and linear computational complexity to achieve spatial weight redistribution through “row-column decoupling”. In its implementation, this mechanism effectively balances the modeling of long-range dependencies with the preservation of local texture features. As a result, it reduces the overall model size while enhancing the model’s capacity to capture essential information in feature maps.
(3) We devise a lightweight and partial faster implementation of cross-stage partial bottleneck with two convolutions (LP-C2f). This module serves as an innovative enhancement over the traditional faster implementation of cross-stage partial bottleneck with two convolutions (C2f [20]) module. By incorporating lighter spatial interaction operators and attention mechanisms, LP-C2f effectively decreases the model size while significantly improving its adaptability to various target shapes and local structures. This advancement enables the model to achieve greater accuracy and enhanced robustness in detecting defects on steel surfaces.

The structure of this study is organized as follows. Section 2 reviews the related work in the field. Section 3 provides an overview of the LESSDD-Net. Section 4 outlines a summary and analysis of the experiment. Finally, Section 5 presents the conclusions drawn from this study and discusses potential avenues for future work.

## 2. Related Work

In recent years, deep learning-based object detection methods have made significant advancements in the detection of defects on steel surfaces [21]. However, in practical applications, the types of defects found on steel surfaces are often complex and varied. These defects not only show considerable scale variations between different classes but also have irregular shapes with indistinct boundaries. Furthermore, they usually manifest as small targets within images that contain significant background interference [22]. As a result, deep learning-based detection of steel surface defects places demanding requirements on current object detection models, which encounter significant challenges related to robustness, generalization, and detection accuracy [23].

In actual industrial production environments, various factors such as changing lighting conditions, surface reflections, and the unique texture characteristics of the workpieces interact and compound, further diminishing overall image quality and significantly complicating defect detection [24]. Additionally, high-precision surface defect detection models that utilize deep learning often have a substantial number of parameters, which creates considerable challenges for deploying these models on mobile devices [25]. To tackle the complex and varied defects found on steel surfaces, researchers have suggested a range of enhancement strategies. These primarily include small object detection, multi-scale feature fusion, and model optimization for lightweight performance, all aimed at achieving effective defect detection.

To effectively tackle the challenge of detecting small targets, Liu et al. [26] proposed the shifted window transformer architecture integrated at the end of the backbone network. They built upon the transformer framework by incorporating a multi-channel feature pyramid network to address the limitations associated with single-channel processing. These modifications significantly improved the model’s capacity to extract global semantic information from small or narrow defects. However, in actual detection tasks, the types of defects encountered are extremely complex and diverse, encompassing not only small defects but also frequently including medium and large defects. Yet the model’s detection accuracy in such scenarios struggles to achieve an ideal level. Ma et al. [27] developed the SSD-YOLO model, specifically tailored for detecting small defects on strip steel surfaces. This model utilizes a dual-branch feature extraction architecture alongside channel-wise feature fusion techniques to improve its ability to capture and represent fine defect characteristics. Additionally, it incorporates a multi-scale high-resolution detection module, enabling accurate segmentation of defect regions. While it achieves a high mean average precision (mAP) on small steel defect datasets, there is still potential for further optimization in detection speed.

Regarding multi-scale feature fusion, Liu et al. [28] developed a parallel architecture utilizing dilated convolutions with different dilation rates to effectively capture contextual information related to multi-scale defects. This approach significantly improves the model’s capacity to detect these flaws. Nonetheless, it still encounters challenges when identifying defects that have irregular shapes, blurred edges, or are obscured by severe background interference. To tackle the prevalent challenges of irregular shapes, significant size variations, and high background similarity in steel surface defects, Ma et al. [29] introduced the position-guided hybrid convolutional neural network and transformer network (PCT-Net), which is based on YOLOv5s. This model creatively combines the strengths of convolutional neural network (CNN) for extracting local details and transformers for modeling global context, allowing for a thorough analysis of complex semantic information from both local and global perspectives. By integrating these approaches, PCT-Net effectively fuses multi-scale features at both local and global levels, facilitating precise defect localization. Experimental results demonstrate that PCT-Net achieves excellent detection performance in tasks related to steel surface defects, exceeding the 30 frames per second (FPS) threshold necessary for industrial applications with a speed of 51.1 FPS. However, there remains room for further optimization and enhancement to better meet the demand for efficient detection in actual production. Building on YOLOv8, Kong et al. [30] introduced an enhanced bidirectional feature pyramid network for the model’s neck structure. This network can perform weighted fusion of multi-scale feature maps, thereby better utilizing feature information across different scales. They also created a novel task-aligned detection head designed to improve performance in both classification and localization tasks. Although the improved model demonstrates excellent detection performance in steel surface defect detection tasks, its large size and high computational demands pose challenges for deployment on mobile devices.

To tackle the challenge of deploying lightweight models, Lin et al. [31] introduced a streamlined version of SI-MobileNet to replace the 101-layer residual network in the deconvolutional single shot detector. This design offers dual advantages: it improves the model’s accuracy in defect detection while also reducing the overall number of parameters. Experimental results indicate that their approach achieves high detection accuracy for minor defects on strip steel surfaces. However, the model’s relatively high computational cost results in detection speeds that do not fully meet practical expectations. Zhou et al. [32] incorporated the VanillaNet module into the YOLOv8 model, allowing it to maintain efficient feature extraction while effectively decreasing the workload on the backbone network. Additionally, they incorporated the advantages of the reparameterized ghost module and large selective kernel modules into the C2f module of YOLOv8, leading to a further reduction in the model’s parameters. Testing results indicate significant reductions in both the model’s parameters and computational costs. However, from the perspective of model optimization, there is still room for further improvement in terms of the number of parameters and computational complexity.

While existing methods have made significant strides in steel surface defect detection, they often face challenges due to their high model complexity [33]. This complexity hinders the deployment of lightweight models, making it difficult for them to function efficiently on resource-limited industrial devices. It also diminishes the practical value and engineering applicability of these algorithms in real industrial environments, creating substantial obstacles to their implementation in production [34]. To achieve a balance between detection accuracy and speed, while addressing the lightweight deployment requirements for steel surface defect detection under resource constraints, we propose a new lightweight algorithm, referred to as LESSDD-Net.

## 3. Methods

### 3.1. Architecture

**Figure 1:**
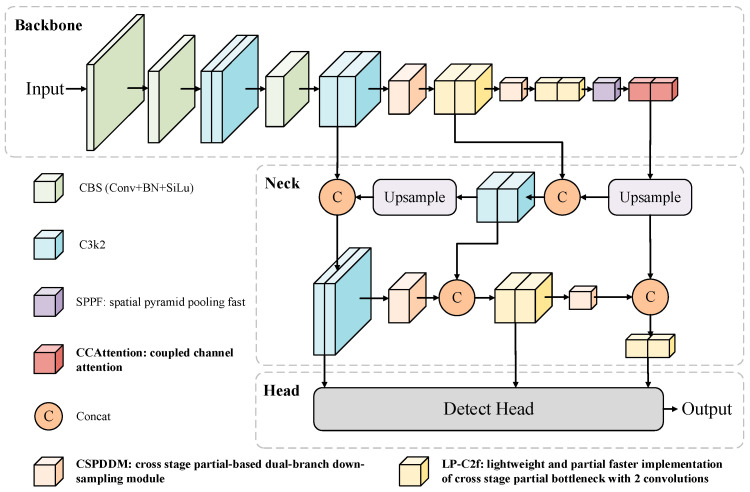
Overall architecture of LESSDD-Net.

As illustrated in Figure 1, the overall architecture of LESSDD-Net comprises three main components, namely, a backbone network, a neck network, and a detection head. First, LESSDD-Net employs CSPDDM in both the backbone and neck networks to improve the feature extraction capabilities of the backbone, leading to enhanced downsampling performance. Additionally, it incorporates the CCAttention mechanism into the backbone network, allowing the model to focus more effectively on critical information in the feature maps while minimizing the impact of irrelevant or redundant data, thereby improving detection performance. In the deeper layers of the network, LESSDD-Net replaces the original C3k2 module found in YOLO11 with the LP-C2f module. This modification reduces the model’s parameter count and computational cost while enhancing its ability to detect multi-scale defects, contributing to lightweight optimization. As a result, the model retains robust performance even in resource-constrained environments.

### 3.2. CSPDDM

**Figure 2:**
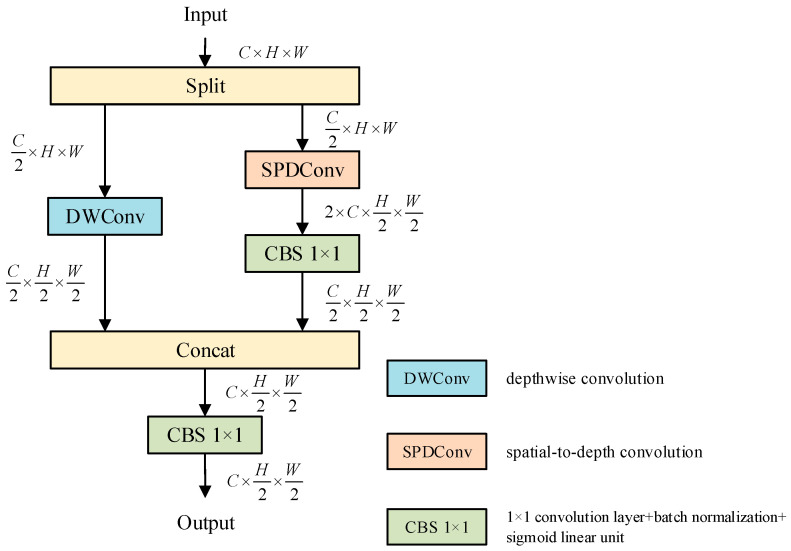
CSPDDM model structure.

CSPDDM is a downsampling module tailored for lightweight networks, effectively balancing computational efficiency with feature representation capabilities. The main concept involves decoupling the processing of spatial and channel information through a dual-branch structure. This approach allows for a reduction in the resolution of feature maps while preserving spatial details and semantic information to the greatest extent possible. The specific structure of CSPDDM is shown in Figure 2.

The CSPDDM module processes the input feature map $X\in {\mathbb{R}}^{C\times H\times W}$ (where C, H, and W refer to the number of channels, height, and width of the feature map, respectively). It begins by evenly splitting the input along the channel dimension into two sub-feature maps, each containing C/2 channels. The first branch extracts local features using depthwise convolution (DWConv [35]) to enhance the model’s ability to perceive spatial information, resulting in a two-fold downsampling of the feature map. Meanwhile, the second branch employs spatial-to-depth convolution (SPDConv [36]) for downsampling, which rearranges the spatial information into the channel dimension, achieving two-fold downsampling without requiring additional parameters. Next, the module modifies the channel count of the sub-feature map using a convolution batch normalization sigmoid linear unit 1 × 1 module (CBS 1 × 1). This process reduces the channel count from 2×C to C/2, ensuring that its dimensions align with the downsampled output from the first branch. Finally, the module concatenates the sub-feature maps from both branches along the channel dimension. This concatenation is followed by fusion through the CBS 1 × 1 module, which enhances the feature map’s nonlinear expressive capability and facilitates interaction between channels. The outcome is a downsampled feature map featuring C output channels and a spatial resolution of $\frac{H}{2}\times \frac{W}{2}$.

The CSPDDM module effectively minimizes computational redundancy and mitigates information loss typically associated with strided convolution or pooling operations in traditional downsampling methods. Its dual-branch architecture preserves lightweight characteristics while substantially improving the capability for multi-scale feature extraction. This makes it particularly well-suited for tasks that require sensitivity to spatial details, such as small object detection, object detection in complex backgrounds, and edge segmentation.

### 3.3. CCAttention

**Figure 3:**
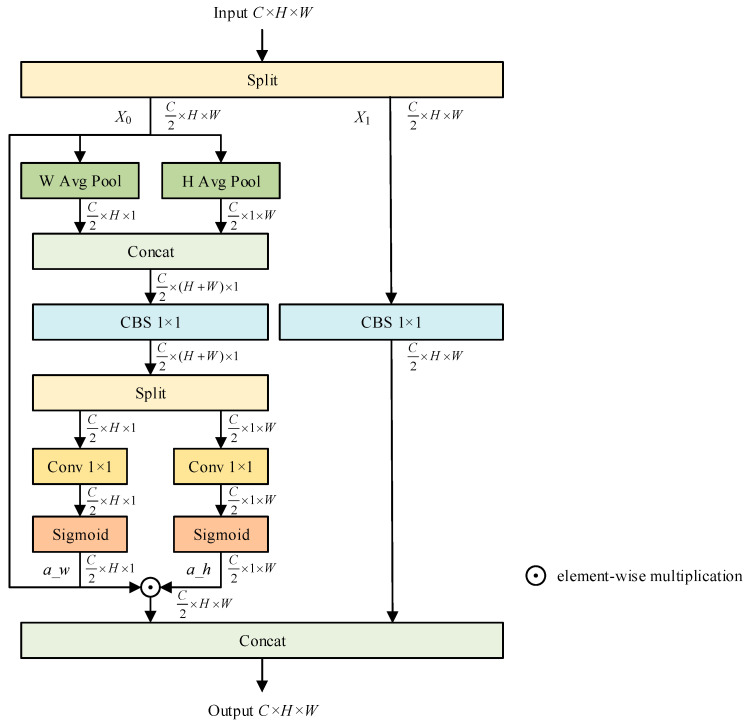
CCAttention structure.

CCAttention is a lightweight, coordinate-aware coupled channel attention mechanism designed to achieve row-column decoupled spatial weight redistribution using zero-space parameters and linear computational complexity. This mechanism effectively balances long-range dependency modeling with the preservation of local texture features. Its structure is illustrated in Figure 3. CCAttention reduces computational load through a channel splitting strategy, reconstructs spatial attention by pixel-wise multiplication of row-column coordinate weights, and retains original information via a residual bypass, thereby mitigating the loss of important features that can occur due to excessive suppression.

For the input feature map $X\in {\mathbb{R}}^{C\times H\times W}$ (where C, H, and W represent the number of channels, height, and width, respectively), CCAttention begins by evenly splitting it along the channel dimension into two identical feature sub-maps, $X_{0}$ and $X_{1}$ ($X_{0},X_{1}\in {\mathbb{R}}^{\frac{C}{2}\times H\times W}$). The sub-map $X_{0}$ is directed to the attention branch for coordinate-aware reweighting, while $X_{1}$ is sent to the residual branch, which undergoes linear mapping through a 1 × 1 convolution. This approach helps preserve shallow texture information and minimizes the risk of excessive suppression of essential features. Utilizing this binary splitting strategy not only halves the model’s computational complexity and significantly enhances computational efficiency but also lays a strong foundation for complementary and synergistic interactions between the dual-path information.

In the attention branch, CCAttention utilizes one-dimensional adaptive average pooling along both horizontal and vertical directions on $X_{0}$ in parallel. This process decouples the two-dimensional spatial information into two complementary one-dimensional coordinate sequences without introducing any learnable parameters, thereby preserving position-sensitive global contextual information. Unlike the scalar global pooling used in standard squeeze and excitation attention, this coordinate decoupling strategy maintains the spatial dimension, preventing complete collapse. As a result, the generated weights can effectively identify salient features in the channel dimension while accurately localizing important regions in the spatial dimension, enabling a joint discriminative analysis of both channel and positional information.

Following this, CCAttention concatenates the two complementary one-dimensional coordinate sequences and inputs the result into the CBS 1 × 1 module for feature fusion. After the fusion process, the output is again divided into two parts, each undergoing processing through independent 1 × 1 convolutional layers and sigmoid activation functions to produce the horizontal attention weight $a\_w\in {\mathbb{R}}^{\frac{C}{2}\times H\times 1}$ and the vertical attention weight $a\_h\in {\mathbb{R}}^{\frac{C}{2}\times 1\times W}$, respectively. This method enables each channel to derive adaptive saliency scores at various spatial positions while maintaining linear computational complexity throughout, ensuring the algorithm remains efficient and scalable.

Once the coupled weights are obtained, the module employs an outer product broadcasting mechanism to perform element-wise multiplication of a_h and a_w, resulting in a two-dimensional attention mask that aligns perfectly with the spatial dimensions of the feature map $X_{0}$. This mask is then applied to the feature map through element-wise matrix multiplication, completing the coordinate-aware feature reweighting. This process facilitates “global-local” dual filtering within the channel dimension. At the global level, different filters are assigned varying importance weights based on the interactions of the coordinate sequences. At the local level, the multiplication of horizontal and vertical weights implicitly captures long-range dependencies, thus preserving and enhancing the details and textures of the feature map.

In the residual branch, $X_{1}$ undergoes a linear transformation solely through the CBS 1 × 1 module. This method cleverly avoids the introduction of additional non-linear factors and effectively preserves the original information, ensuring that it is not overly suppressed. This approach maintains the stability and accuracy of information transmission. Ultimately, the features $X_{0}$ processed by the attention branch are concatenated with $X_{1}$ from the residual branch along the channel dimension, restoring the output feature channel count to match that of the input. The resulting output features encapsulate globally important information selected by attention while retaining the original, unsuppressed representations, leading to efficient and nuanced feature enhancement.

### 3.4. LP-C2f

The motivation for the lightweight bottleneck with partial convolution (LBPC) stems from a thorough analysis of the redundancy inherent in 3 × 3 convolution operations found in standard bottleneck module. In the conventional design, the bottleneck structure first compresses the input features to half their original channel count through a 1 × 1 convolution. It then utilizes a 3 × 3 convolution to facilitate the interaction of spatial information, followed by another 1 × 1 convolution to revert the feature maps to their original channel dimension. Although this “compress-expand” strategy achieves robust accuracy, it becomes increasingly inefficient as the channel count grows. The computational requirements of the 3 × 3 convolution escalate quadratically with the number of channels, leading to a substantial concentration of parameters that tightly couple global channels. This scenario places a significant burden on edge processors, particularly in terms of memory bandwidth and computational resources.

**Figure 4:**
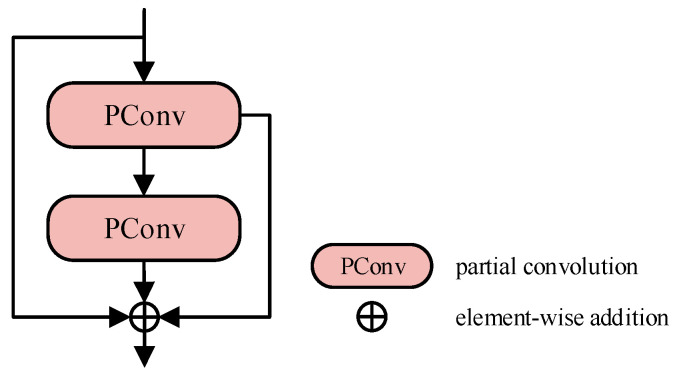
LBPC model structure.

To overcome this limitation, we introduce LBPC, a lightweight alternative with the architecture detailed in Figure 4. In this design, the input features are initially processed by the first group of partial convolutions (PConv [37]), which applies a 3 × 3 convolution to only 1/4 of the channels, while the remaining 3/4 of the channels are preserved through identity mapping. The output from this stage is then passed to a second group of PConv, which again convolves just 1/4 of the channels to achieve further feature refinement. Finally, the original input feature map, along with the outputs from both stages, are combined using element-wise addition to generate the final output. This approach retains the gradient backpropagation paths and the nonlinear expressive capabilities of the standard bottleneck module, while reducing the number of multiplication operations to just 1/16 of the original count. Consequently, it significantly decreases memory access frequency and offers a lightweight atomic operator suitable for the subsequent reconstruction of macro-scale architectures.

**Figure 5:**
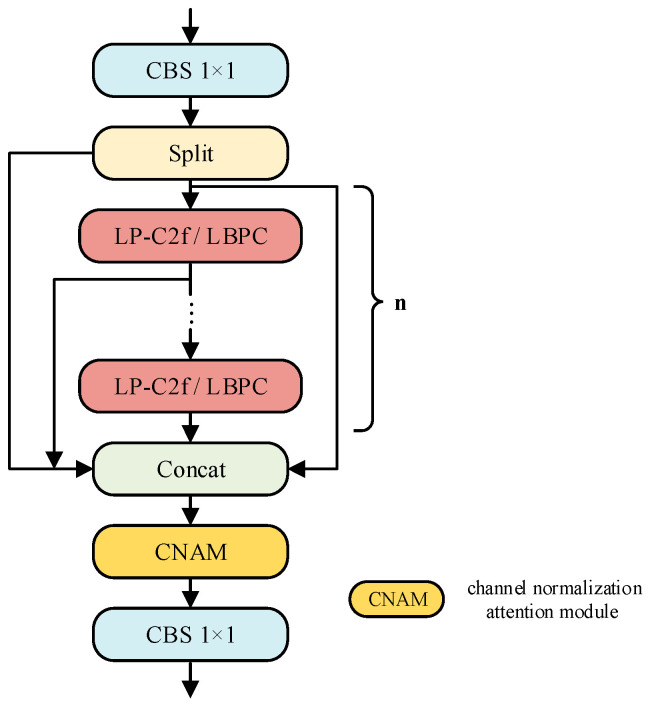
LP-C2f model structure.

Following the development of the lightweight spatial interaction operator, we introduce the LP-C2f module, illustrated in Figure 5. LP-C2f adopts the macro-architecture of C2f but replaces the standard bottleneck module entirely with LBPC modules along the feature extraction path. The input features initially pass through a CBS 1 × 1 module for dimensionality reduction and are then divided into two branches along the channel dimension. One branch functions as a cross-stage shortcut, allowing it to bypass all intermediate layers directly. In contrast, the other branch dynamically selects multiple LP-C2f or LBPC modules through a selectable discriminative parameter *n*, refining the spatial information of the feature maps in a half-channel complementary manner. However, concatenating outputs from multiple branches can create conflicts in scale and semantics, leading to potential information overload during direct fusion. To mitigate this issue, LP-C2f concatenates all intermediate results from the various sub-paths along the channel dimension. These concatenated outputs are then processed by the channel normalization-based attention module (CNAM [38]), which emphasizes critical information in the feature maps while reducing background noise. Notably, CNAM adds minimal additional parameters and computational overhead, significantly enhancing the recall ability of subsequent detection heads for medium and small targets. Finally, the feature maps are adjusted back to the target channel count using a 1 × 1 convolution.

LP-C2f preserves the gradient reuse advantage of C2f while replacing the high-density 3 × 3 convolutions found in the standard bottleneck structure with a more lightweight PConv. Additionally, it integrates a lightweight CNAM during feature fusion. This design facilitates efficient model detection while achieving dual compression of both parameter count and computational complexity. The module can be seamlessly integrated into any YOLO-style backbone network or feature pyramid architecture, effectively substituting the original C2f or C3k2 structures without the need to alter loss functions, label assignment strategies, or training hyperparameters.

## 4. Experiment

### 4.1. Dataset

The dataset utilized in this study is the Northeastern University surface defect database for detection (NEU-DET [39]), which is publicly accessible and provided by Northeastern University. This dataset encompasses six common types of surface defects found in hot-rolled steel strips: crazing (CR), inclusion (IN), patches (PA), pitted_surface (PI), rolled_in_scale (RO), and scratches (SC). Each image in the dataset is a 200 × 200 pixel grayscale image, with a total of 300 images for each defect type, resulting in an overall total of 1800 images.

**Figure 6:**
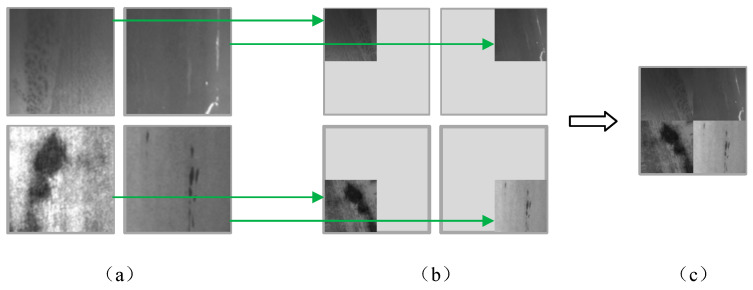
Mosaic data augmentation example. (**a**) are the input images; (**b**) are the images obtained by flipping, scaling, rotating, and altering the color gamut of the input images, followed by proportionally random cropping; (**c**) is the image obtained after mosaic data augmentation.

To tackle the challenging conditions associated with defect detection in hot-rolled steel, this study utilizes mosaic data augmentation [40] to effectively increase the sample size and enhance the model’s generalization capabilities. Mosaic augmentation creates a composite image by integrating semantic information from four original images, as illustrated in Figure 6. The process begins by randomly selecting four original images, as depicted in Figure 6a. Each selected image is then subjected to various transformations, including flipping, scaling, rotation, and color space adjustments, followed by random proportional cropping. Subsequently, the cropped images are arranged into four quadrants: top-left, top-right, bottom-left, and bottom-right, as shown in Figure 6b. The final step involves concatenating these arranged images into a single composite image, as represented in Figure 6c.

The augmented dataset comprises 1200 images for each defect class, resulting in a total of 7200 images. The dataset is divided into training, validation, and test sets in an 8:1:1 ratio. After this division, the training set contains 5760 images, while both the validation and test sets consist of 720 images each.

### 4.2. Training Environment and Parameter

Experiments are carried out using the PyTorch framework to train and evaluate the proposed model. The experimental setup utilizes Ubuntu 20.04.5 as the operating system, along with CUDA 11.8, PyTorch 1.11.0, and Python 3.8.20. The hardware configuration featured an Intel (R) Xeon (R) Platinum 8474C CPU, an RTX 4090D GPU with 24 GB of VRAM, and 80 GB of RAM.

The input image size is standardized to 640 × 640 pixels. For model parameter optimization, we utilize the stochastic gradient descent optimizer to iteratively update the parameters. The training is conducted over 150 epochs with a batch size of 32. To promote efficient convergence during training, we implement a cosine annealing strategy for dynamic adjustment of the learning rate. The initial learning rate is set to 0.01, and a weight decay mechanism with a coefficient of 0.0005 is incorporated to mitigate overfitting and improve model generalization.

### 4.3. Evaluation Metrics

This study uses average precision (AP), mean average precision (mAP), frames per second (FPS), the number of model parameters (Params), and floating point operations (FLOPs) to provide a thorough evaluation of LESSDD-Net’s performance.

mAP serves as a key metric for evaluating the overall performance of models in object detection tasks. It is computed by calculating the arithmetic mean of the average precision for all categories, as detailed in the formula below:

(1) $$mAP=\frac{1}{C}\sum_{i=1}^{C}AP_{i},$$

where C denotes the number of defect categories in the NEU-DET dataset. AP is defined as follows:

(2) $$AP=\int_{0}^{1}P(r)dr,$$

where r denotes the recall rate. P(r) represents the precision at a specific recall rate r.

FPS indicates how many image frames a model can process within one second. Higher FPS values reflect faster detection speeds and better real-time performance. Params directly determine the memory required for the model’s storage and runtime execution. FLOPs, meanwhile, is a core metric for assessing computational cost, intuitively reflecting the total floating-point operations needed for the model’s forward propagation. Generally, smaller Params and FLOPs values mean a lighter model.

### 4.4. Ablation Experiments

To objectively and clearly assess the enhancements provided by each module in LESSDD-Net compared to the baseline model YOLO11n, and to validate its effectiveness in detecting surface defects on hot-rolled steel strips, we conducted eight systematic ablation experiments. The specific protocols for these experiments are outlined in Table 1. Case 1 serves as the baseline model, YOLO11n. Cases 2, 3, and 4 integrate CSPDDM, CCAttention, and LP-C2f into the baseline model, respectively. Cases 5, 6, and 7 further combine CSPDDM with CCAttention, CSPDDM with LP-C2f, and CCAttention with LP-C2f, respectively, within the baseline model. Finally, Case 8 represents the fully integrated LESSDD-Net model proposed in this study.

The results of the ablation experiments are shown in Table 2. Case 1 corresponds to the baseline model YOLO11n, with 2.58 M Params, 6.3 G FLOPs, 137.39 FPS, and mAP of 73.04%. Case 2 introduces the CSPDDM module into the baseline model. Compared to the baseline, it shows a 19.38% reduction in Params, an 11.11% decrease in FLOPs, a 2.83% increase in FPS, and a 1.30% improvement in mAP. These performance improvements demonstrate that adding the CSPDDM module effectively reduces model complexity while enhancing runtime efficiency and detection accuracy. Case 3 incorporates the CCAttention module. Compared to the baseline, it exhibits an 8.91% decrease in Params, a 3.17% reduction in FLOPs, a 4.13% increase in FPS, and a 1.01% improvement in mAP. This result suggests that CCAttention can optimize the model structure to some extent and enhance its performance. Case 4 uses the LP-C2f module. Due to its nested structure, FPS decreases slightly compared to the baseline; however, mAP improves by 1.42%, while Params and FLOPs decrease by 11.63% and 6.35%, respectively.

Clearly, each of the three modules has its own advantages. To further enhance model performance, we attempted to combine the modules in pairs. For Case 5, incorporating both CSPDDM and CCAttention simultaneously results in significant decreases in model Params and FLOPs, with both FPS and mAP improved, and outperforms the performance of using either module alone. Specifically, compared to the original model, Params and FLOPs decrease by 28.29% and 14.29%, respectively, while mAP and FPS increase by 2.27% and 5.78%, respectively. For Case 6, after using CSPDDM with LP-C2f, compared to Case 4, which uses only LP-C2f, Params decrease by 21.93%, FLOPs reduce by 11.86%, FPS improves by 1.32%, and mAP increases by 1.36%. This comparative result indicates that the combination of CSPDDM and LP-C2f further optimizes model performance, excelling in reducing complexity and enhancing accuracy. Similarly, for Case 7, incorporating CCAttention with LP-C2f results in a 10.09% decrease in Parameters, a 1.69% reduction in FLOPs, a 2.16% improvement in FPS, and a 0.39% increase in mAP compared to Case 4. These performance improvements suggest that the combination of CCAttention and LP-C2f also enhances model performance to a certain extent.

Finally, Case 8 represents a comprehensive optimization of the YOLO11n model through the integration of the three previously mentioned modules. Although the FPS slightly declined compared to the baseline model, the mAP reached 75.37%, which is the highest among all the ablation experiment cases. Additionally, the Params and FLOPs were the lowest across all groups, recorded at 1.55 million and 5.0 billion, respectively. When compared to the baseline YOLO11n model, LESSDD-Net demonstrates a 3.19% improvement in mAP, a 39.92% reduction in Params, and a 20.63% decrease in FLOPs.

**Figure 7:**
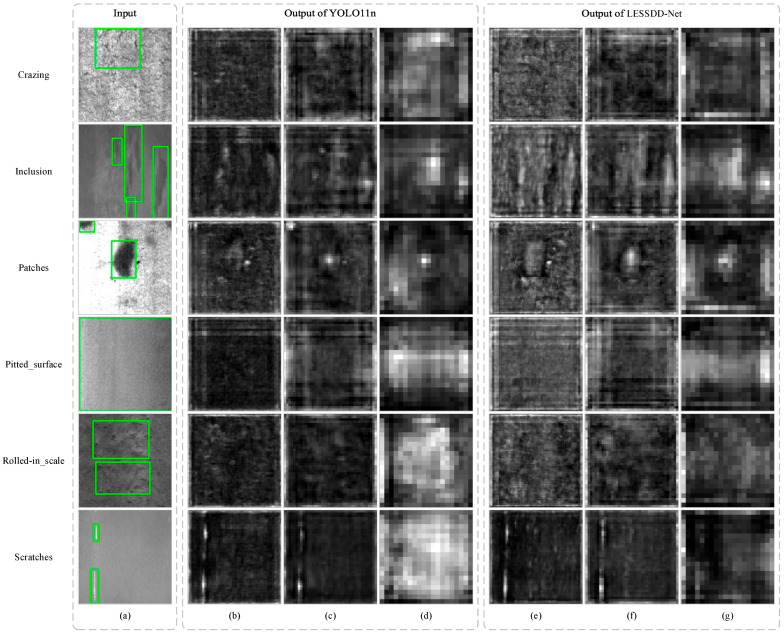
Visualization results of average feature maps extracted by YOLO11 and LESSDD-Net. (**a**) is the input image; (**b**–**d**) are three sets of feature maps output by YOLO11 (from left to right, P2, P3, and P4 layers); (**e**–**g**) are three sets of feature maps output by LESSDD-Net.

In this section, we perform a visualization analysis of the feature maps extracted by LESSDD-Net and YOLO11n to validate the effectiveness of LESSDD-Net. The results of this visualization are shown in Figure 7, where the average feature map is calculated by averaging all channels of the feature map.

A comparison of the 6 sets of average feature maps reveals that those produced by LESSDD-Net contain richer textural details, with this improvement being particularly evident in Figure 7f. This enhancement stems mainly from the integration of the CSPDDM downsampling module, which markedly strengthens the model’s capability to capture multi-scale features from the feature maps, thereby retaining more fine-grained information. Furthermore, the incorporation of CCAttention enables LESSDD-Net to focus more accurately on defect-prone regions of steel surfaces, effectively suppressing background noise and yielding more discriminative feature representations.

### 4.5. Comparison Experiments

Table 3 reports the experimental results of LESSDD-Net alongside eight mainstream lightweight object detection models on the augmented NEU-DET dataset. Compared with these models, LESSDD-Net achieves the lowest number of Params and FLOPs, while delivering the highest mAP. Overall, LESSDD-Net surpasses the current state-of-the-art lightweight object detection models, with an average improvement of 5.30% in mAP, a 28.26% increase in FPS, a 73.53% reduction in Params, and a 60.71% decrease in FLOPs.

In the comprehensive evaluation, LESSDD-Net demonstrates superior performance over both YOLOv5n and YOLOv8n in all metrics except detection speed. Specifically, when compared with YOLOv5n, LESSDD-Net achieves a 2.60% increase in mAP, alongside reductions of 38.00% in Params and 29.58% in FLOPs. In comparison to YOLOv8n, LESSDD-Net delivers a 2.18% improvement in mAP, with parameters reduced by 48.50% and FLOPs reduced by 38.27%. Compared with YOLOv10n, which operates at a similar FPS, LESSDD-Net increases mAP by 5.47%, while reducing Params and FLOPs by 42.59% and 39.02%, respectively, thereby achieving higher detection accuracy with lower resource consumption. Furthermore, in comparison with YOLOv12n, which features relatively low computational cost, LESSDD-Net delivers a 3.92% improvement in mAP and an 81.11% increase in FPS, along with reductions of 38.25% in Params and 13.79% in FLOPs. These performance gains ensure efficient detection while simultaneously enhancing accuracy and minimizing computational overhead.

In conclusion, LESSDD-Net achieves an effective balance between detection accuracy, computational cost, and processing speed, making it well-suited for the practical requirements of lightweight deployment in steel surface defect detection.

**Figure 8:**
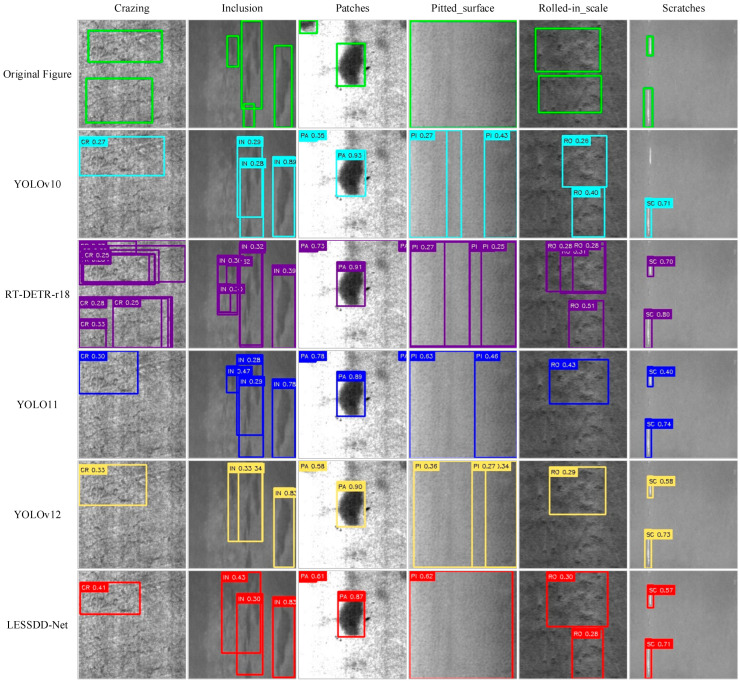
Detection performance of different models on the augmented NEU-DET dataset.

To provide a more intuitive illustration of the improvements brought by LESSDD-Net, partial detection results from various models on the augmented NEU-DET dataset are presented in Figure 8. The analysis reveals that, in crazing defect detection, RT-DETR-r18 frequently produces duplicate detections, while YOLOv10, YOLOv11, YOLOv12, and LESSDD-Net generally exhibit missed detections. However, despite occasional missed detections, LESSDD-Net achieves the highest prediction confidence among models of the same category. For inclusion defects, RT-DETR-r18 again suffers from duplicate detections, whereas YOLOv10 tends to miss detections. In the case of patches defects, both RT-DETR-r18 and YOLOv11 produce false positives. When detecting pitted_surface defects, YOLOv10, RT-DETR-r18, YOLOv11, and YOLOv12 all show varying levels of duplicate detections. For rolled_in_scale defects, RT-DETR-r18 produces duplicate detections, while YOLOv11 and YOLOv12 fail to detect some instances. In scratch defect detection, YOLOv10 exhibits missed detections. In contrast, LESSDD-Net demonstrates notable advantages by accurately classifying and localizing defects, while maintaining consistently high prediction confidence, thereby ensuring reliable performance across diverse defect categories.

**Figure 9:**
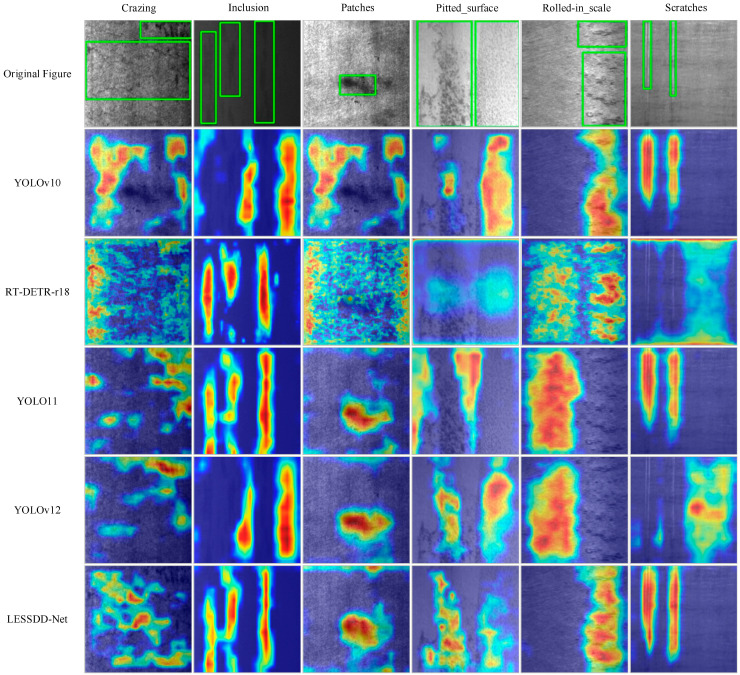
Heatmaps of different models on the augmented NEU-DET dataset.

To further assess the performance of the proposed LESSDD-Net, the eigen class activation mapping method was applied to generate heatmaps, enabling a visual comparison of different models. The heatmap results for each model are displayed in Figure 9. The analysis shows that RT-DETR-r18 demonstrates limited feature attention capability, with its feature distribution appearing disorganized and irregular. When processing inclusion and patches defects, YOLOv10 tends to focus primarily on regions surrounding the target rather than the defect itself. Similarly, for pitted_surface and rolled_in_scale defects, YOLOv11 concentrates on areas adjacent to the target. YOLOv12 exhibits a comparable tendency, focusing on the surrounding regions in the detection of inclusion, rolled_in_scale, and scratch defects. In contrast, LESSDD-Net, leveraging the robust feature extraction capability of LP-C2f along with the effective feature enhancement provided by CCAttention, accurately directs its attention to defect targets while substantially minimizing focus on irrelevant background information. This targeted attention improves both the precision and reliability of defect detection.

### 4.6. Generalization Experiments

Without loss of generality, we compare LESSDD-Net with several mainstream object detection models from recent years using the SSGD dataset, which is designed for defect detection on smartphone screen glass [46]. This dataset comprises a total of 2504 images focused on identifying surface defects on smartphone screens. We randomly partition the dataset into training, validation, and test sets in an approximate ratio of 8:1:1. Consequently, the training set includes 2004 images, while both the validation and test sets consist of 250 images each.

The experimental results of LESSDD-Net compared to other object detection models on the SSGD dataset are presented in Table 4. The data indicates that LESSDD-Net, as proposed in this study, outperforms mainstream object detection models from recent years by achieving the highest R value and mAP score, along with the lowest number of parameters and computational demands. Additionally, LESSDD-Net exhibits commendable detection speed, being only slightly slower than YOLOv8n, which has the fastest detection speed. Specifically, in comparison to contemporary object detection models, LESSDD-Net shows an average increase of 18.19% in R value and 15.55% in mAP, alongside an average reduction of 4.58 million parameters and 11.28 GFLOPs in computations. Furthermore, its detection speed is improved by an average of 28.71%. These results demonstrate that LESSDD-Net effectively maintains robust detection performance in smartphone screen surface defect detection tasks while minimizing both parameter count and computational load.

**Figure 10:**
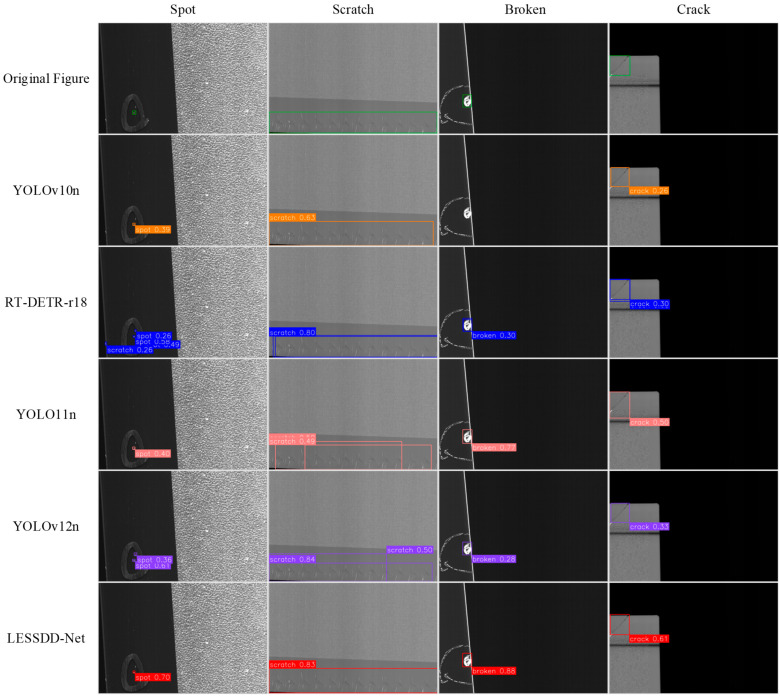
Detection results of different lightweight models on the SSGD dataset.

Partial detection results of different lightweight models on the SSGD dataset are shown in Figure 10. It is not difficult to see that there are obvious false detection phenomena in the RT-DETR-r18 model in the detection of Spot defect categories. For scratch defect categories, the three models of RT-DETR-r18, YOLOv11n and YOLOv12n are prone to duplicate detection problems. YOLOv10n is prone to missed detection issues on broken defect categories. RT-DETR-r18 is also prone to duplicate detection problems in the crack defect category. Compared with other models, LESSDD-Net shows more significant advantages. It can not only complete the task of defect classification and positioning in a high-precision way, effectively avoid common problems such as missed detection and repeated detection, but also always maintain a high confidence level of the prediction box, showing excellent performance.

## 5. Conclusions

To overcome the limitations of existing steel surface defect detection models, which are often bulky and difficult to deploy effectively on mobile devices, this study proposes a novel lightweight detection framework, LESSDD-Net. The model integrates the CSPDDM lightweight downsampling module and the LP-C2f feature extraction module, enabling efficient extraction of key feature map information while markedly reducing both parameter count and computational complexity. In addition, CCAttention is embedded into the backbone network to strengthen the model’s ability to concentrate on crucial regions within the feature maps, thereby enhancing the precision and reliability of defect-related information capture.

Experimental results on the augmented NEU-DET dataset show that LESSDD-Net achieves a detection accuracy of 75.37%, with only 1.55 M Params and a computational complexity of 5.0 G. Relative to the baseline model YOLO11n, LESSDD-Net increases mAP by 3.19%, while reducing parameter count by 39.92% and lowering computational complexity by 20.63%. These results highlight the model’s ability to deliver improved detection performance while maintaining a lightweight architecture suitable for resource-constrained deployment.

Compared to current mainstream object detection models, LESSDD-Net not only achieves higher detection speeds but also demonstrates significant advantages in detection accuracy, model parameter count, and computational complexity, achieving optimal performance across all compared models. Specifically, LESSDD-Net attains, on average, a 5.30% improvement in mAP, a 73.53% reduction in Params, and a 60.71% decrease in computational complexity, highlighting its strong competitiveness and suitability for efficient, lightweight deployment. The experiments conducted on the public SSGD dataset emphasize the robust generalization capability of the LESSDD-Net model.

Although LESSDD-Net has achieved notable lightweight optimization in steel surface defect detection tasks, it still encounters certain challenges when dealing with defects that exhibit irregular shapes and indistinct boundaries, or are affected by severe background interference. Under these conditions, both detection accuracy and processing speed leave space for further improvement. In future research, efforts will focus on two main directions: performance enhancement and model compression. Potential strategies include employing knowledge distillation and neural architecture search techniques, aiming to develop a defect detection model that maintains exceptional accuracy and robustness while remaining as lightweight as possible.

## Figures and Tables

**Table 1:** Ablation experiment schemes.

Schemes	CSPDDM	CCAttention	LP-C2f
Case 1			
Case 2	√		
Case 3		√	
Case 4			√
Case 5	√	√	
Case 6	√		√
Case 7		√	√
Case 8	√	√	√

**Table 2:** Comparison of ablation experimental results.

Schemes	Params/M	FLOPs/G	FPS	mAP/%	AP_CR_/%	AP_IN_/%	AP_PA_/%	AP_PI_/%	AP_RO_/%	AP_SC_/%
Case 1	2.58	6.3	137.39	73.04	48.14	70.17	82.77	94.84	49.10	93.24
Case 2	2.08	5.6	141.28	73.99	51.97	67.37	84.52	94.77	53.48	91.82
Case 3	2.35	6.1	143.06	73.78	48.33	69.62	84.19	95.57	54.65	90.29
Case 4	2.28	5.9	125.75	74.08	50.99	69.44	82.32	94.90	55.83	91.01
Case 5	1.85	5.4	145.33	74.70	48.21	68.60	87.59	94.90	57.83	91.04
Case 6	1.78	5.2	127.41	75.09	50.77	71.72	85.65	96.18	54.51	91.72
Case 7	2.05	5.8	128.47	74.37	46.87	73.10	87.18	95.10	54.13	89.82
Case 8	1.55	5.0	131.09	75.37	51.77	70.10	84.05	94.29	61.10	90.89

**Table 3:** Comparison of experimental results on the augmented NEU-DET.

Model	Params/M	FLOPs/G	FPS	mAP/%
YOLOv5n [41]	2.50	7.1	165.11	73.46
MobileNet-SSD [42]	8.80	5.3	30.28	69.26
RTMDET-ting [43]	4.88	8.0	72.50	69.09
YOLOv8n [20]	3.01	8.1	153.65	73.76
YOLOv10n [44]	2.70	8.2	124.51	71.46
RT-DETR-r18 [45]	19.86	53.0	61.82	70.01
YOLO11n [18]	2.58	6.3	137.39	73.04
YOLOv12n [14]	2.51	5.8	72.38	72.53
LESSDD-Net	1.55	5.0	131.09	75.37

**Table 4:** Experimental results of different models on SSGD.

Models	P/%	R/%	mAP/%	Parameters/M	FLOPs/G	FPS
YOLOv8n	60.78	35.17	37.20	3.01	8.1	157.01
YOLOv10n	31.17	33.77	32.35	2.70	8.2	117.05
RT-DETR-r18	39.71	34.09	35.35	19.86	53	25.56
YOLO11n	56.84	33.00	31.20	2.58	6.3	127.12
YOLOv12n	44.04	25.87	28.98	2.51	5.8	47.93
LESSDD-Net	37.25	38.27	38.15	1.55	5.0	133.16

## Data Availability

The NEU-DET is available online at http://faculty.neu.edu.cn/songkechen/zh_CN/zdylm/263270/list/index.htm (accessed on 25 November 2025).

## References

[1] Fan J., Wang M., Li B., Liu M., Shen D. (2024). ACD-YOLO: Improved YOLOv5-based method for steel surface defects detection. IET Image Process..

[2] Zhang W., Huang T., Xu J., Yu Q., He Y., Lai S., Xu Y. (2025). DF-YOLOv7: Steel surface defect detection based on focal module and deformable convolution. Signal Image Video Process..

[3] Xia K., Lv Z., Zhou C., Gu G., Zhao Z., Liu K., Li Z. (2023). Mixed receptive fields augmented YOLO with multi-path spatial pyramid pooling for steel surface defect detection. Sensors.

[4] Xie W., Ma W., Sun X. (2025). An Efficient Re-parameterization Feature Pyramid Network on YOLOv8 to The Detection of Steel Surface Defect. Neurocomputing.

[5] Liang X., Li Y., Wang X., Liu P., Shen Y., Guo J. (2025). Adaptive shape imitation and selective semantic guidance for industrial surface defect detection. Expert Syst. Appl..

[6] Zheng X., Liu W., Huang Y. (2025). Legendre multiwavelet-based feature attention guidance lightweight network for accurate steel surface defect classification. Eng. Appl. Artif. Intell..

[7] Jiang P., Xu Z., Fan W., Zhang J. (2025). An On-line Global-local Defect Detection Framework for Wide Cold-rolled Strip Steel. Eng. Appl. Artif. Intell..

[8] Zhang L., Hu Y., Tan R., Zeng W., Chen J. (2025). Lightweight train image fault detection model based on location information enhancement. Eng. Appl. Artif. Intell..

[9] Zhang T., Pan P., Zhang J., Zhang X. (2024). Steel surface defect detection algorithm based on improved YOLOv8n. Appl. Sci..

[10] Zabin M., Kabir A.N.B., Kabir M.K., Choi H.-J., Uddin J. (2023). Contrastive self-supervised representation learning framework for metal surface defect detection. J. Big Data.

[11] Lv Z.L., Zhao Z.Q., Xia K.W., Gu G.J., Liu K., Chen X.L. (2024). Steel surface defect detection based on MobileViTv2 and YOLOv8. J. Supercomput..

[12] Liu Q., Liu M., Jonathan Q., Shen W. (2024). A Real-time Anchor-free Defect Detector with Global and Local Feature Enhancement for Surface Defect Detection. Expert Syst. Appl..

[13] Ren S., He K., Girshick R., Sun J. (2016). Faster R-CNN: Towards real-time object detection with region proposal networks. IEEE Trans. Pattern Anal. Mach. Intell..

[14] Tian Y., Ye Q., Doermann D. (2025). Yolov12: Attention-centric real-time object detectors. arXiv.

[15] Liu W., Anguelov D., Erhan D., Szegedy C., Reed S., Fu C.-Y., Berg A.C. SSD: Single Shot Multibox Detector. Proceedings of the European Conference on Computer Vision.

[16] Wang H., Liu J., Zhao J., Zhang J., Zhao D. (2025). Precision and speed: LSOD-YOLO for lightweight small object detection. Expert Syst. Appl..

[17] Zhang L., Chen J., Chen J., Wen Z., Zhou X. (2024). LDD-Net: Lightweight printed circuit board defect detection network fusing multi-scale features. Eng. Appl. Artif. Intell..

[18] Khanam R., Hussain M. (2024). Yolov11: An overview of the key architectural enhancements. arXiv.

[19] Zhou W., Sun X., Qian X., Fang M. (2025). Asymmetrical Contrastive Learning Network via Knowledge Distillation for No-Service Rail Surface Defect Detection. IEEE Trans. Neural Netw. Learn. Syst..

[20] Varghese R., Sambath M. Yolov8: A novel object detection algorithm with enhanced performance and robustness. Proceedings of the 2024 International Conference on Advances in Data Engineering and Intelligent Computing Systems (ADICS).

[21] Li Y., Han Z., Wang W., Xu H., Wei Y., Zai G. (2024). Steel surface defect detection based on sparse global attention transformer. Pattern Anal. Appl..

[22] Wei Y., Wang R., Zhang M., Wang Y., Zhou F., Bian X. (2025). Ade-yolo: Real-time steel surface flaw recognition through enhanced adaptive attention and dilated convolution fusion. Signal Image Video Process..

[23] Yan R., Zhang R., Bai J., Hao H., Guo W., Gu X., Liu Q. (2023). STMS-YOLOv5: A lightweight algorithm for gear surface defect detection. Sensors.

[24] Li X., Fan Z., Liu Q., Wan X. (2025). DSP-YOLO: An improved YOLO11-based method for steel surface defect detection. Meas. Sci. Technol..

[25] Shao Y., Ning J., Shao H., Zhang D., Chu H., Ren Z. (2024). Lightweight face mask detection algorithm with attention mechanism. Eng. Appl. Artif. Intell..

[26] Liu H., Chen C., Hu R., Bin J., Dong H., Liu Z. (2024). CGTD-net: Channel-wise global transformer-based dual-branch network for industrial strip steel surface defect detection. IEEE Sens. J..

[27] Ma C., Li Z., Xue Y., Li S., Sun X. (2025). High-frequency dual-branch network for steel small defect detection. Arab. J. Sci. Eng..

[28] Liu R., Huang M., Gao Z., Cao Z., Cao P. (2023). MSC-DNet: An efficient detector with multi-scale context for defect detection on strip steel surface. Measurement.

[29] Ma Y., Zhang Z. (2025). Position-Guided Hybrid Convolutional Neural Network and Transformer Network for steel strip surface defect detection. Eng. Appl. Artif. Intell..

[30] Kong S., Kong Y., Chi X., Feng X., Ma L. (2025). A TBD-YOLO-Based Surface Defect Detection Method for Hot Rolled Steel Strips. Russ. J. Nondestruct. Test..

[31] Lin L., Wen A., Wang Y., Zhao S., Zhang S., Yan J., Zhou Y., Zhou W. (2024). Surface defect detection of strip steel based on GT-CutMix augmentation algorithm and improved DSSD model. Meas. Sci. Technol..

[32] Zhou H., Zhang Y., Yan C. (2024). Lightweight strip steel surface defect detection algorithm based on YOLOv8-VRLG. J. Electron. Imaging.

[33] Li C., Wen Z., Huang H., Mo H., Zhou S., Zhu Z. (2025). An efficient lightweight detection model for steel surface defects with dynamic deformable head. Eng. Res. Express.

[34] Wang Y., Sun H., Luo K., Zhu Q., Li H., Sun Y., Wu Z., Wang G. (2025). A lightweight YOLOv11-based framework for small steel defect detection with a newly enhanced feature fusion module. Sci. Rep..

[35] Chollet F. Xception: Deep Learning with Depthwise Separable Convolutions. Proceedings of the IEEE Conference on Computer Vision and Pattern Recognition.

[36] Sunkara R., Luo T. No More Strided Convolutions or Pooling: A New CNN Building Block for Low-resolution Images and Small Objects. Proceedings of the Joint European Conference on Machine Learning and Knowledge Discovery in Databases.

[37] Liu G., Reda F.A., Shih K.J., Wang T.-C., Tao A., Catanzaro B. Image inpainting for irregular holes using partial convolutions. Proceedings of the European Conference on Computer Vision (ECCV).

[38] Zhang H., Lu Z., Chen X., Lu S., Yao L. (2025). Masked contrastive generative adversarial network for defect detection of yarn-dyed fabric. J. Supercomput..

[39] Bao Y., Song K., Liu J., Wang Y., Yan Y., Yu H., Li X. (2021). Triplet-Graph Reasoning Network for Few-shot Metal Generic Surface Defect Segmentation. IEEE Trans. Instrum. Meas..

[40] Gai R., Chen N., Yuan H. (2023). A detection algorithm for cherry fruits based on the improved YOLO-v4 model. Neural Comput. Appl..

[41] Liu G., Hu Y., Chen Z., Guo J., Ni P. (2023). Lightweight object detection algorithm for robots with improved YOLOv5. Eng. Appl. Artif. Intell..

[42] Meng J., Jiang P., Wang J., Wang K. (2022). A mobilenet-SSD model with FPN for waste detection. J. Electr. Eng. Technol..

[43] Tang Y., Wang Y., Wang X., Oh J., Si G. (2025). Automated scene-adaptive rock fragment recognition based on the enhanced segment anything model and fine-tuning RTMDet. Rock Mech. Rock Eng..

[44] Wang A., Chen H., Liu L., Chen K., Lin Z., Han J. (2024). Yolov10: Real-time end-to-end object detection. Adv. Neural Inf. Process. Syst..

[45] Zhao Y., Lv W., Xu S., Wei J., Wang G., Dang Q., Liu Y., Chen J. Detrs Beat Yolos on Real-time Object Detection. Proceedings of the IEEE/CVF Conference on Computer Vision and Pattern Recognition.

[46] Han H., Yang R., Li S., Hu R., Li X. SSGD: A smartphone screen glass dataset for defect detection. Proceedings of the ICASSP 2023—2023 IEEE International Conference on Acoustics, Speech and Signal Processing (ICASSP).

